# Supplementary material for: Chitosan/Pomegranate Seed Oil Emulgel Composition as a New Strategy for Dermal Delivery of Hydrocortisone
Source: Int J Mol Sci. 2024 Mar 28;25(7):3765. doi: 10.3390/ijms25073765 (PMC11012218; doi:10.3390/ijms25073765)
Supplement: Supplementary file 1 [file ijms-25-03765-s001.zip › ijms-2916079-supplementary.pdf]

SUPPLEMENTARY MATERIAL

Article

Chitosan/Pomegranate Seed Oil Emulgel Composition As a New Strategy for Dermal Delivery of Hydrocortisone

Zofia Helena Bagińska [1], Magdalena Paczkowska-Walendowska [2], Anna Basa [3], Michał Rachalewski [4], Karolina Lendzion [4], Judyta Cielecka-Piontek [2] and Emilia Szymańska [5,*]

[1] Department of Pharmaceutical Technology, Student Scientific Group, Medical University of Bialystok, Mickiewicza 2c, 15-222 Białystok, Poland; baginska.zosia@gmail.com
[2] Department of Pharmacognosy and Biomaterials, Poznan University of Medical Sciences, Rokietnicka 3 Str., 60-806 Poznań, Poland; e-mail: mpaczkowska@ump.edu.pl (M.P-W.); jpiontek@ump.edu.pl (J.C-P.)
[3] Faculty of Chemistry, University of Bialystok, Ciołkowskiego 1K, 15-245 Białystok, Poland; E-mail: abasa@uwb.edu.pl (A.B.)
[4] Dr Irena Eris, Centre for Science and Research, Armii Krajowej 12, 05-500 Piaseczno, Poland; e-mail: michal.rachalewski@drirenaeris.com (M.R.); karolina.lendzion@drirenaeris.com (K.L.)
[5] Department of Pharmaceutical Technology, Medical University of Bialystok, Mickiewicza 2c, 15-222 Białystok, Poland; e-mail: emilia.szymanska@umb.edu.pl (E.S.)
* Correspondence: emilia.szymanska@umb.edu.pl; Tel.: +48-8574-856-16 (E.S.)

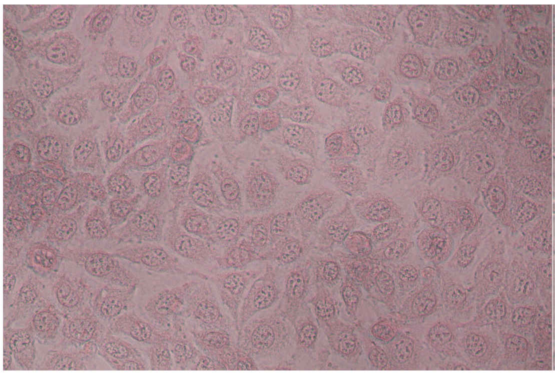

(a)

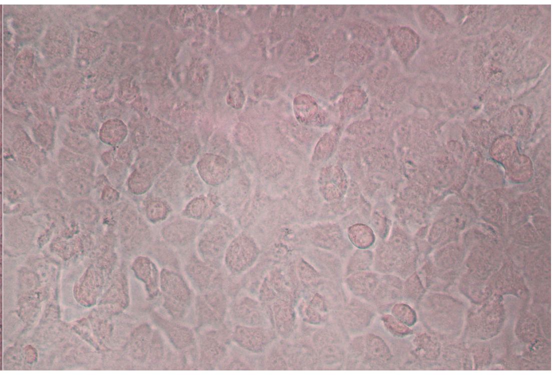

(b)

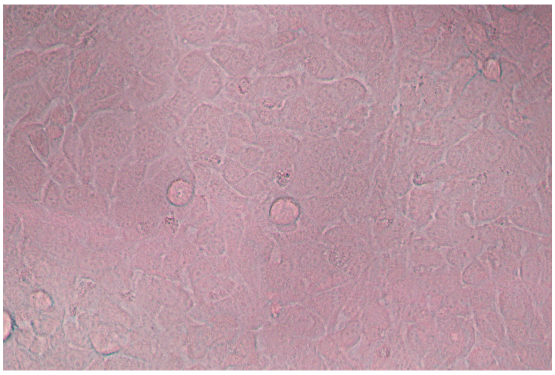

(c)

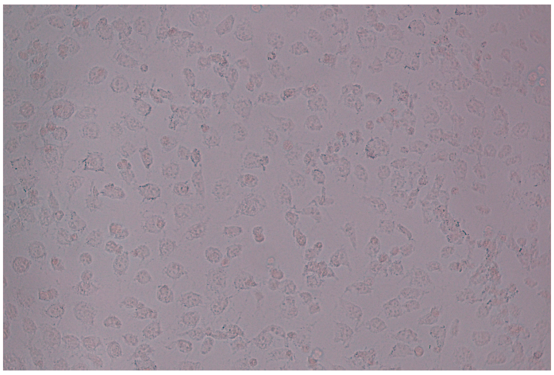

(d)

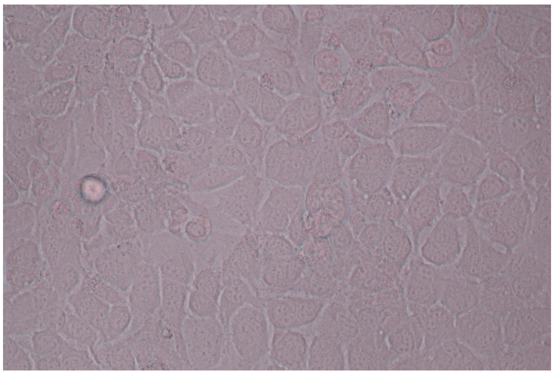

(e)

Figure S1: Representative images of mouse fibroblast NCTC 929 cells after 24h incubation with emulgels: (**a**) E-1; (**b**) E-2; (c) E-3 and controls: (**d**) PC – 3% SDS, (**e**) NC – PBS; original magnification ×200.
